# Deep mechanism design: Learning social and economic policies for human benefit

**DOI:** 10.1073/pnas.2319949121

**Published:** 2025-06-16

**Authors:** Andrea Tacchetti, Raphael Koster, Jan Balaguer, Liu Leqi, Mîruna Pislar, Matthew M. Botvinick, Karl Tuyls, David C. Parkes, Christopher Summerfield

**Affiliations:** ^a^Google DeepMind, London EC4A 3TW; ^b^Princeton Language and Intelligence, Princeton University, Princeton, NJ 08544; ^c^Gatsby Computational Neuroscience Unit, University College London, London W1T 4JG, United Kingdom; ^d^Yale Law School, Yale University, New Haven, CT 06520-8215; ^e^Harvard John A. Paulson School Of Engineering And Applied Sciences, Harvard University Boston, MA 02138; ^f^Department of Experimental Psychology, University of Oxford, Oxford OX1 3UD, United Kingdom; ^g^UK AI Safety Institute, London SW1A 2AS, United Kingdom

**Keywords:** mechanism design, deep learning, game theory

## Abstract

Human society is coordinated by mechanisms that control how prices are agreed, taxes are set, and electoral votes are tallied. The design of robust and effective mechanisms for human benefit is a core problem in the social, economic, and political sciences. Here, we discuss the recent application of modern tools from AI research, including deep neural networks trained with reinforcement learning (RL), to create more desirable mechanisms for people. We review the application of machine learning to design effective auctions, learn optimal tax policies, and discover redistribution policies that win the popular vote among human users. We discuss the challenge of accurately modeling human preferences and the problem of aligning a mechanism to the wishes of a potentially diverse group. We highlight the importance of ensuring that research into “deep mechanism design” is conducted safely and ethically.

The human species has prospered by acquiring sophisticated forms of social organization (1). The creation of a rich cumulative culture, advanced technologies, and a globalized economy have been made possible by the development of mechanisms that allow knowledge to be disseminated, resources to be exchanged, policy decisions to be coordinated, and behavioral norms or laws to be enforced. For example, economic policies set taxation or determine how public goods are distributed between richer and poorer members of society, education allows efficient knowledge dissemination from experts to novices, media networks allow ideas to be shared widely and instantly, democracy permits preference aggregation over the population either directly or via elected representatives, and justice systems encourage people to obey social norms. The nature of extant social mechanisms, the principles by which they should be designed, and their consequences for human society are core research themes in the social, economic, and political sciences.

In economics, mechanism design is often studied with structural models, which capture economic processes using a formal, analytic framework (a system of equations), often with a focus on the equilibria that may emerge as agents interact (2). Social exchange is often cast as a game in which one player (the *principal* or *social planner*) sets the rules of exchange for other agents (3–6). Given a resource to be allocated (such as an asset being sold or a public good being distributed) and the differing preferences of the agents over that resource, the goal of the mechanism designer is to find a set of rules that achieve some desired outcome—usually related to the efficiency of the exchange or the maximization of utility across agents under a given social choice function. Mechanism design is challenging because agents’ preferences are private, and so the principal cannot optimally allocate resources by fiat. Instead, agents disclose their preferences strategically and are not obliged to tell the truth. For example, consider the case where two buyers with different preferences compete for an asset by making a single sealed bid. A mechanism that awards the asset (at a predetermined price) to highest bidder will encourage agents to overstate their preferences; by contrast, a mechanism that sets the sale price at the value of the winning bid encourages agents to understate their preferences. Both thus promote dishonest revelation of preferences and encourage inefficient outcomes. However, under a mechanism that obliges the highest bidder to pay the price offered by the second highest bidder,^*^ rational agents are incentivized to accurately report their preferences (7). The field thus attempts to design mechanisms (such as this “second price” auction) that (under strong assumptions) offer irrefutable guarantees of honest price setting and efficient exchange. Similar principles have been applied to a range of settings, including the design of clearing houses that allow organs to be matched with donors, and applicants allocated efficiently to their preferred school, college, or training program (8).

This framework has been widely used to design auctions, in which assets such as physical goods, advertising space, or broadcasting bandwidth are sold (9). Using structural models, optimal principles have been derived analytically for auctions involving a single asset and multiple potential buyers whose preferences adjust iteratively as other bids are revealed (10). However, for more complex problems, hand-designing mechanisms with optimality guarantees rapidly become intractable. In the case of auctions, even a generalization to two bidders and a two-item bundle remains only partially solved (11). Formulated as a linear program (12), the number of decisions and constraints grows exponentially with the granularity of the value function, rapidly requiring prohibitive levels of computation (13). For a broader range of problems—from designing fair electoral systems to setting optimal tax policies—further problems arise. For example, voting systems based on strictly ordinal (ranked) preferences over options are beset by impossibility results showing that strategic behavior is inevitable unless other undesirable properties (e.g., restricted options, dictatorship, or intransitive preference cycles) are permissible. Optimal taxation policies depend on multiple factors including the productivity distribution over the population and relative preferences for consumption and leisure. Similarly, for double-sided auctions— those involving multiple sellers as well as buyers—efficiency necessarily trades off with incentive compatibility, individual rationality, and budget balance (14). In these cases, finding a theoretically optimal solution becomes a seemingly impossible constraint satisfaction problem.

In many settings, mechanisms of exchange may create a *social dilemma* in which the interest of the individual and those of the group are placed in tension (15, 16). In these cases, mechanisms may succeed by exploiting human preferences or biases that promote cooperative behaviors or by sanctioning those who choose to defect. For example, people may have a bias to prefer that resources are distributed uniformly over players, even where players have not contributed equally (17, 18). More generally, real-world decisions may be noisy and idiosyncratic or boundedly rational (subject to unknown but stereotyped constraints) such that human behavior deviates significantly from that predicted by rational agent models. For example, optimal decisions in multiplayer games may require recursive inference about the preferences of other agents, whereas humans may use myopic or “level-k” cognition (19). It may thus be necessary to model human behavior, or better understand human preferences, to design successful economic mechanisms, curtailing the feasibility of structural modeling approaches.

To address these problems, economists have used *agent-based models* (ABMs) as a numerical tool for studying the dynamics of exchange among simulated agents (humans or firms) acting according to stylized policies (20–22). By releasing theorists from the need to derive closed-form solutions, ABMs allow the exploration of more sophisticated scenarios with more realistic agents, including those with boundedly rational policies (23). For example, a major class of ABM uses evolutionary game theory to study the emergence of norms, institutions, or coalitions in social groups (24), and ABM has also been deployed to study mechanism design (25, 26), including using game theoretic methods, with a focus on identifying mechanisms for double-sided auction design (27). However, most traditional ABM approaches, including game theory, can only be used to study relatively low-dimensional policies in stylized environments. Typically, thus, modelers must decide by hand how to parameterize the social planner’s policy so that mechanism design remains constrained by the imagination of the researcher.

In this review, we discuss another alternative: the use of new methods from machine learning and AI research to design social, political, and economic mechanisms (12, 28). Deep neural networks are powerful, expressive tools for solving complex optimization problems and are increasingly being deployed to identify new economic solution concepts: an emerging field newly referred to as “differentiable economics” (28). Unlike game theoretic approaches, deep learning models can explore an almost unbounded set of policies, allowing them to be applied to high-dimensional environments such as video games (29). This opens new frontiers for modeling economic behavior, including the use of more human-realistic agents. Of course, the increased expressiveness of deep RL agents often comes at the cost of interpretability and transparency, and so it is important for researchers to identify those problems where the application of more complex models is warranted.

One significant branch of research has used multiagent systems in the form of deep networks trained with reinforcement learning (RL) to study the emergence of social phenomena, such as cooperation or reputation formation, in spatiotemporally complex environments (30–34). This has yielded surprising new insights, such as the observation that the existence of “silly rules”—social norms with no obvious purpose—fosters the learning of socially compliant policies among group members, in turn yielding collective benefits (35). Here, however, we focus on the use of machine learning systems for mechanism design. We first consider the promising use of deep networks for auction design. In subsequent sections, we consider applications in which neural networks are trained with data collected from people playing economic games, including social dilemmas, and used to act as a social planner, designing mechanisms that are tailored to satisfy desirable outcomes, for example, by maximizing equality, aggregate return, or the approval rates (votes) of human users.

## Deep Learning for Auction Design

Auctions have been used to sell goods or services since antiquity, but their formal study is a more recent phenomenon. For several decades, before the advent of deep learning, auction design played a prominent role in economics. Seminal results such as the Vickrey–Clarke–Groves (VCG) auction family, that ensures efficient allocation and truthful bidding (7, 36, 37), or Myerson’s optimal auction, which allows sellers to maximize their revenue (10), laid the foundations of modern auction theory.

An auction proceeds in three stages. During bidding, buyers first report their ranking or valuations for items or bundles to the auctioneer via a specified means (such as open outcry, popular in art auctions, or sealed bid, often used to sell property). The auctioneer then allocates items to bidders according to a specified protocol (such as highest bid wins). Finally, the auctioneer computes payment according to a given rule (such as the buyer paying the second highest price bid). Deep neural networks have been used to design each of these three stages—*bidding*, *allocation,* and *payment*—to satisfy an objective such as maximizing (or minimizing) auctioneer revenue while retaining the truthfulness and efficiency of the auction.

To model the behavior of buyers and sellers in an auction, we need to quantify their preferences over possible outcomes. Traditionally, it has only been possible to express preferences over a discrete and finite (although potentially large) set of alternatives, such as the complete set of possible ways that five items could be allocated to 3 bidders. One important way that deep learning has been used in auction design is to additionally allow value functions over continuous domains (38–41). For example, if bundles include continuously varying quantities of multiple different goods, then the function that maps bundles onto preferences for each participant in the auction is potentially complex and high-dimensional. However, neural networks are differentiable and can approximate continuous, high-dimensional functions. These two properties allow their application to the design of efficient allocation protocols and compact “bidding languages.” For example, bidders can communicate the weights of a neural network approximator of their preference over a continuous domain such as location, which is far more practical than specifying a valuation for each point in space. Moreover, these representations lend themselves to gradient-based allocation protocols, where individual or aggregate welfare can be maximized with off-the-shelf optimization algorithms, opening the door to VCG-like mechanisms for continuous domains (42).

VCG has been among the most popular auction mechanisms for settings with strategic bidders and multiple items because it provides guarantees of truthfulness (people are incentivized to bid their true valuation) and efficiency (goods are allocated to those who desire them most). However, a fundamental result, known as the Gibbard-Satterthwaite theorem, states that when designing auctions, these two desirable properties inevitably come at the cost of strong budget balance, which is the requirement the auction should not generate a surplus for the auctioneer (this result, which means that auctions must either cost money or be susceptible to manipulation, follows from Arrow’s impossibility theorem). Budget balance is often sought in settings where the focus is on efficient allocation, for example when governments sell licenses to build public works such as hospitals or parks. Thus, a major problem in auction design has been to find ways to redistribute excess contributions back to participants in ways that do not undermine VCG efficiency or strategy proofness. Most attempts to reduce the aggregate payments and increase efficiency curtail the generality of the VCG mechanism. Neural networks have been used to address this problem, by learning elaborate redistribution functions that minimize aggregate payments without compromising the general applicability of the mechanism (43, 44).

Neural networks have also been used to solve the converse problem in auction design, namely, to maximize the revenue of the auctioneer. While this problem was solved for the single-item case more than 40 y ago, optimal designs for two or more items remain elusive. A deep learning model called RegretNet implements both payment and allocation functions as neural networks and recovers provably optimal designs in known settings, as well as maximizing revenue beyond the state-of-the-art in cases where provably optimal mechanisms are not known (13).

Another major application for deep networks in auction design is to model settings where the same buyers and sellers may interact repeatedly. This incurs fundamentally different challenges to the one-shot auction case because new equilibria emerge when participants can condition their bids on past as well as current information. In some cases, these may damage the competitiveness of the auction, for example by opening the door to collusion or other forms of strategic rigging. Methods from deep RL can be used to model the behavior of bidders in repeated auctions. For example, using deep Q-learning, one paper found that violations of independence across repeated auctions, and emergence of collusive behaviors were more common in first-price than second-price auctions. First-price auctions incentivize participants to outbid their opponents by just one bid increment, which facilitates recoordination on low bids after if repeated experimentation among the same bidders is possible. However, providing information about the lowest bid to win increases the competitiveness of first-price auctions, going some way to mitigate the problem (45).

## Learning to Set Optimal Taxes and Incentivize Cooperation

Many forms of social organization require cooperation among self-interested agents. A well-known social dilemma (46) occurs when a rivalrous public good that self-replenishes at a fixed rate is freely available to multiple individuals for private benefit; each individual must temper their consumption to maintain the resource at stable levels (for example, by limiting catch sizes to maintain sustainable fish stocks). Conceived as a game playing out over time horizons of unknown length, social dilemmas often have multiple Nash equilibria—including those that are Pareto-efficient (e.g., where everyone cooperates and sustains the commons) and Pareto-deficient (e.g., where everyone overconsumes for private benefit, and the commons is tragically depleted to collective detriment). This implies that a social planner might be able to design mechanisms that are more likely to identify Pareto-efficient solutions by drawing upon situations in which humans are able to overcome social dilemmas. Indeed, human cultures often endogenously develop social norms that enforce limited consumption, for example by establishing behavioral norms for rights to grazing, fishing, or irrigation (47).

One role for the planner in a social dilemma may be to provide incentives or penalties to players to encourage them toward more collaborative policies. Indeed, human players in economic games will pay to sanction defecting colleagues and when the game mechanism permits them to do so, leading to increases in cooperation (48). In one study, a machine social planner was trained with RL to emit costly signals that rewarded or penalized players depending on their responses in an iterated version of the two-player matrix game Prisoner’s Dilemma. Interestingly, the planner first learned to reward cooperation, but as cooperation spread through the environment, and this became more costly, it switched to sanctioning defection—a mixed policy that strongly promoted cooperative behavior in the other agents (49). In another study, a simple AI system was inserted into a social network in which humans were making decisions to cooperate or defect in an economic game played with their neighbors. The AI increased overall levels of cooperation in the network relative to baseline (50). In a related study, an AI system that used a fixed policy to help engineer the network structure, by recommending disengagement with partners uncooperative partners, similarly increased human cooperation (51). The use of more powerful AI systems trained with deep learning may yield further benefits in shaping social networks to maximize human cooperation, but the use of opaque algorithms to shape human interactions is also risky, as it may inadvertently create undesirable side effects, such as the polarized opinions found on social media.

Deep RL has also been used to design more complex mechanisms in a multiagent setting. Optimal tax theory describes the problem of setting a tax policy (under a given social welfare function) in order to provision public goods while minimizing economic distortions, such as a disincentive to work (52). Closed-form theories of tax-setting model grossly simplified settings, such as a single (or two) cycles of revenue generation, and make strong assumptions about income elasticity (agent sensitivity to tax policy). To address these shortcomings, one ambitious study (53) sought to discover a tax policy that jointly maximized productivity and equality for agents playing a spatiotemporal (video) game called Gather-Trade-Build. Artificial agents (players) received income for building houses from gathered or purchased wood or stone in a simulated 2D world and sought to maximize their expected discounted isoelastic utility (posttax income minus labor) (54). Individual players were differentially rewarded for building (depending on their *skill*) and thus labor specialization into builders (high skill) and gatherers (who sold wood and stone to builders using a double-price auction; low skill) was an emergent property of the game. Players paid taxes in a yearly cycle, which are redistributed equally in a lump sum by a social planner, whom the authors call the “AI Economist.” The planner’s job was to set the taxation brackets on income level in a way that maximizes the product of productivity (sum of pretax income) and equality (inverse Gini coefficient for income).

This sophisticated environment allowed the authors to examine the tax policy learned by the planner and compare it to real-world alternatives, such as the US Federal progressive tax schedule or the free market (no taxes). Strikingly, the AI Economist learned to recreate a well-known theory that proposes optimal tax rates as a function of income distribution and elasticity of income (55). Plausible market dynamics emerged in the simulation (for example, taxing the richer players led to a drop in commodity prices, which in turn reduced income for those with lower build skill) and simulated agents even learned tax-gaming strategies such as moving income between yearly cycles. In a subsequent study, the AI Economist was used to model the interplay between economic output and public health during the Covid-19 pandemic, in an environment governed by a susceptible-infectious-recovered (SIR) model of disease spread and a plausible macroeconomic model linking lockdown-incurred unemployment to economic output. The AI economist captured empirically observed real-world dynamics (such as a strong but declining stringency of response to the pandemic) and identified a policy proposal that was predicted to minimize fatalities without undue decline in employment (56).

More recently, deep RL has been deployed to explore the costs and benefits of different policy interventions in other economic settings, including studying the interplay of labor, production and consumption in macroeconomic models (57), the efficiency and resilience of the economy to shocks under different design principles for market-driven platforms (such as Uber and Amazon) (58), and the heuristic remuneration policies (and their impact on employee welfare) that emerge when a principal agent (wage-setter) is boundedly rational (59). Together, these studies show that deep RL can be used to study the consequences of different policy interventions in complex, quasi-naturalistic economies, taking ABM a step closer to the real world. As modeling studies can simulate a broad range of economic scenarios, including those that have not occurred in the real world, they have the potential to address a well-known critique often leveled at work in empirical economics, that there are no plausible counterfactual data with which to falsify a theory (60). However, if deep RL (or any ABM) is used to generate these data, the validity of the results is always bounded by the plausibility of the model and the reliability of its fit to the data.

## Modeling Citizen Preferences

However, one perennial issue for ABM is whether simulated agents adequately capture the behavior of humans in real-world economies. A failure to do so will lead to implausible results. In the original AI Economist study, the resulting social plan involved much higher marginal tax rates (in excess of 50% for all income levels) than most governments are willing to consider, and moreover, taxation was even higher (up to 80%) for those on lower incomes, a regressive policy that seems unlikely to be politically palatable.^†^ In other work, optimal tax rates may vary over a wide range (~20 to ~80%) depending on differing assumptions about elasticity of income (55). In particular, divergence between simulated (agent-based) players and humans may arise because humans are boundedly rational and tend to use heuristic policies for decision-making that are efficient but not strictly utility-maximizing, or exhibit cognitive biases, such as overweighting losses relative to gains or being sensitive to the framing of a prospect (61, 62).

One solution to this problem is to directly measure human preferences, for example, by conducting experiments with crowdsourced participants. In some environments, such as video games, collecting human data can be a means to enhance agent performance through expert demonstrations (63), or to harness our intuitions about the game to provide agents with strategic shaping rewards (64, 65). When training agents to play cooperative video games with people, actively modeling human behavior has been shown to help (66), but training against a sufficiently diverse AI population may be an alternative solution (67). In the domain of natural language processing (NLP), the modeling of human preferences over text-based outputs has become a standard feature of fine-tuning pipelines for large language models, helping improve the quality of summarization, the accuracy of question answering or the fidelity of translation (68–70). Learning directly from human preferences offers an opportunity for AI systems to act in a way that is aligned with our values, ideally making them more helpful and less harmful (71, 72).

Thus, if we wish ABMs of social planning to translate to the real world, we need to accurately model the responses of human agents to the choices made by the mechanism designer. This is a challenge because human preferences are complex, and people’s responses to automated systems are not always rational (73) and may differ sharply from those elicited by human-designed mechanisms (74, 75). One important approach is the use of participatory methods, in which stakeholders are surveyed to identify the variables that may be important for the planner to optimize against. In combination with a voting rule from social choice theory (such as Borda score, a tally over voters’ ranked preferences) this can be used to combine human preferences into a single prescription for the planner’s objective (bearing in mind that self-reported human preferences may not always match those revealed by behavior). This approach has been used in settings as variable as the design of algorithms for matching charitable donations to recipients (76), for prioritizing transplant patients for receipt of kidneys (77), and for machine decisions in fictitious moral conundrums involving autonomous vehicles (78).

In one recent study, a variant of this approach in combination with deep RL was used to train an AI system to directly maximize human preferences over redistribution mechanisms in an economic game based on a classic social dilemma known as the (iterated) linear public goods game (PGG) (79). In the game, a group of players each receive a monetary endowment and decide over repeated rounds whether to keep it for private consumption or contribute it to a common pool for public benefit (a multiplier is applied to public funds before they are redistributed back to players). In the standard version of the game (80), players receive endowments of equal value, and public funds are redistributed uniformly back to players (an egalitarian policy). Empirically, human participants playing the game under these conditions rapidly learn that contributing little or nothing to the public fund (free riding on the generosity of others) maximizes their return, and so public funds tend to rapidly collapse over rounds (81).

Here, however, the authors elaborated the game in two important ways. First, player endowments could be unequal; in some games, there was a single *head* player (more advantaged) who received 10 coins on each round, and three *tail* players (less advantaged) who received $2\leq e_{\mathrm{tail}}\leq 10$ coins. Second, the redistribution policy could be learned by a machine social planner, who was a deep neural network trained with RL to directly maximize the preferences of players in the game. Preferences were measured by asking participants to experience two successive games involving different mechanisms (AI and baseline) and vote for the one that they preferred; votes were aggregated with a simple majority rule. The question asked was can the AI system design a redistribution mechanism that maximizes stated human preferences (akin to winning votes in an election based on policy implementation), and if so, what properties does the preferred mechanism have?

In the case where all players receive the same endowment ($e_{\mathrm{tail}}=10$), redistributing the public fund back to players in proportion to their contributions is a Pareto-dominant mechanism for rational agents with full information. This effectively turns the public good into a private good, allowing each player to simply recoup their contributions with interest via a series of parallel one-player games. Empirically, under this *libertarian* mechanism human players rapidly learn to play the Nash strategy—to make full contributions to the common pool, in contrast with the steep decline in public funding observed under *egalitarian* redistribution (82). In this setting, the deep RL agent learned a policy overcompensated public giving, presumably so that participants (who had no *a priori* knowledge of the mechanism) learned the optimal strategy faster. In doing so, it received more votes from humans than a strictly libertarian baseline.

However, the setting where initial endowments are unequal poses a more interesting challenge for the social planner. Here, more *libertarian* redistribution exacerbates inequality because the advantaged player can contribute (and thus receive) larger sums than those with small initial endowments players; this policy is thus dispreferred by the less well-off majority. However, more *egalitarian* redistribution funnels the contributions of the player with the largest endowment into the pockets of the less advantaged players, which disincentivizes that player from contributing, leading to a general collapse of the public fund. Thus, the AI has to find a compromise between overtaxing the rich (which lowers productivity) and an unfettered free market (which advantages only a minority). It achieved this by learning to implement a policy with two salient features. First, it redistributed funds in proportion to contributions normalized by endowment—a policy known to political philosophers as “liberal egalitarian” because it takes into account their initial opportunity as well as their contribution to society (83). Second, it learned to sanction those whose relative public contributions were low in value, by withholding return; in doing so, it incentivized players to give at least half of their endowment to the public fund. Thus, the AI system learned a hybrid policy that combined ideas from opposite ends of the political spectrum (a progressive redistribution policy based on opportunity, combined with a stern tendency to sanction free riders). The mechanism it designed received more votes than a range of baselines devised by the researchers and was also more popular than policies that were directly implemented by other human participants playing the role of social planner, when evaluated on a novel group of players (79).

## Methodological Challenges for Deep Mechanism Design

When using deep learning to design new social and economic mechanisms, a number of methodological issues arise. One significant problem is due to the joint nonstationary dynamics of learning in the planner (who refines the mechanism) and the players (who respond to the mechanism). If planner and players coadapt with comparable learning rates, then at any given time the mechanism will be optimized for players’ behavior in the past but not the present, rendering learning potentially unstable. This is one instance of the more general problem of opponent shaping, in which agents learning together coadapt to each other’s policies in mutually detrimental ways (like when two pedestrians collide because they move together to avoid each other). In multiagent research, past approaches to this challenge include population-based training (84) or allowing agents privileged access to each other’s learning signals (gradients) (32). Another solution to this problem is known as *bilevel optimization*, in which the planner and players are trained in nested loops, with (perennially resampled) players adapting rapidly to a given social policy in an “inner loop,” but the policy itself being trained over many batches of different players in a slower “outer loop.” One recent paper showed that bilevel optimization during training allows the planner to learn a policy that “shepherds” simulated coplayers toward desirable stable outcomes to iterated normal form games, such as Prisoner’s Dilemma, Battle of the Sexes, and Stag Hunt. In the economic game requiring redistribution described above, a fully differentiable bilevel optimization pipeline learned a mechanism that encouraged contributions to the public fund in simulated coplayers. This policy transferred to novel groups of human participants playing the game for real incentives under comparable conditions (85).

A second problem concerns how to best model human responses to the mechanism. Collecting human data is time-consuming and expensive, so training the social planner with people directly “in the loop” is rarely feasible. Instead, in ref. 79, the authors first collected a large body of data from participants playing the game against baseline mechanisms, and then trained neural network models (LSTMs) to imitate human play, including their voting policy, using behavioral “cloning.” The AI social planner was then optimized to maximize the votes of these ‘cloned’ players in a fully simulated pipeline, which could be arbitrarily scaled, before transferring the learned mechanism successfully back to new human players. Successfully imitating human play in multiagent settings is a challenge in itself (86), but here the difficulty is exacerbated because the human players being imitated were necessarily trained under a different (and potentially suboptimal) mechanism. In fact, this is a general paradox for human-centered mechanism design: that good human models are needed to learn a successful mechanism, but the mechanism itself is needed to train those models.

To address this problem, one recent paper proposes an iterative approach in which the social planner is gradually trained over multiple rounds of human data collection and imitation learning (87). Using the economic redistribution game described above, the authors began by collecting votes from human players exposed to two successive instances of a *random* redistribution mechanism $\pi_{0}$. They then cloned the human data and used the simulated players to train a new mechanism $\pi_{1}$ that would (in expectation) maximize votes relative to those already experienced. Two instances of this second-generation mechanism were then deployed with a new group of human participants, and again votes were recorded, and the whole process was repeated. The idea was that intrinsic stochasticity in choice would drive preferences apart for the two instantiations of the mechanism, providing a gradient that the planner could use to incrementally refine the mechanism. After ~7 cycles, this process led to the creation of a mechanism $\pi_{n}^{*}$ that was more popular than strong baseline social planning policies conceived by humans. The technique, dubbed “human-centered mechanism design zero” (or HCMD-zero), has the merit of avoiding all need for researcher intuitions about what might make a good mechanism (e.g., to design baseline rival mechanisms for training) but instead relies entirely on human data to iterate toward an optimal solution. Interestingly, related methods (involving repeated cycles of data collection, imitation learning, and model refinement) have recently been used to train a deep network “adversary” that tries to undermine human choices in a bandit task (88) or a deep network that can successfully synthesize images that mislead neurons in the macaque visual cortex into generating strong responses to otherwise nonpreferred stimuli (89). Initiating the social planner with a policy that is theoretically optimal (for a rational agent) rather than random might warm-start the design process and allow for faster iteration toward a mechanism that is maximally effective for people (90).

A third challenge arises from the presence of strategic participants. Machine learning enables mechanism design in very general settings, including those with human participants whose behavior might deviate from full rationality in significant ways, or involve repeated interactions, and learning-induced nonstationarity. However, neither the resulting mechanisms nor the learning process itself is guaranteed to be robust to strategic behavior. For example, it may well be possible to successfully train a participant that is able to significantly disrupt a mechanism trained with the “optimal” mechanisms derived with machine learning that are described above, such as human-centered mechanism design. This notion is similar to that of adversarial robustness in vision classifiers (91). Furthermore, there is no guarantee that the process of constructing the mechanism itself is robust to adversarial attacks, for example, if participants know their behavior will be used in training, they may attempt to sway the final policy of the mechanism. This second-order consideration is peculiar to behavioral mechanism design and has no direct parallel in other learning applications such as computer vision or language models.

Finally, when modeling human participants’ responses to mechanism choices, designers must select between imitation-based or incentive-based learning. Imitation-based approaches use supervised learning to predict participants’ behavior and learn to leverage the structure exposed in previous interactions to extrapolate to new circumstances. Incentive or equilibrium-based learning on the other hand aims at reconstructing, in full or in part, the underlying utility function of each participant. Behavior predictions are then made on the assumption that participants act in their own self-interest. Both methods suffer from identifiability issues, and their performance outside of their training distribution can be quite poor. In particular, imitation-based approaches will not account for any equilibrium effects, where the behavior of the group goes from one equilibrium to the next (e.g., rate of telephone adoption has a step change once enough people buy a telephone); similarly incentive-based approaches suffer from identifiability issues where out of equilibrium strategies are hardly observed, making it hard to fully characterize participants’ preferences (92).

## Ethical Challenges for Deep Mechanism Design

One distinctive feature of deep mechanism design is the need to identify a mechanism that is preferred by a group of people. This challenge of “group alignment” poses a unique conundrum: When stakeholders disagree, how do we choose which policy to adopt? For example, when AI is trained to directly maximize human preferences, researchers must decide which groups to sample, and which social welfare function to choose to aggregate preferences. In ref. 79, the authors used a popular crowdsourcing platform and imposed a simple majority rule, which runs the risk of disenfranchising those with legitimate minority views—the so-called “tyranny of the majority” (93). Sampling carefully chosen stakeholders (76), developing sophisticated voting rules (94) or using machine learning to help with sortition (selecting representatives in an unbiased way) (95) are all steps toward a more considered framework for fair and democratic group alignment. Ultimately, however, settings where AI systems are trained from human votes or numerical shaping rewards only a limited version of democracy, which overlooks the crucial role of deliberation, institutions, and representative leadership (96). One hope is that advances in NLP may help people find agreement not just by voting but by deliberating in natural language, and recent work points in this direction (97).

Another related challenge for deep mechanism design is that social policies gain legitimacy by being transparent and interpretable. The outputs of deep networks, especially large-scale architectures trained with RL, are often hard to interpret, and so offer only weak guarantees that any mechanisms designed will be explainable to human users (or other people who are affected by resulting externalities). In the studies described above, the authors variously attempt to tackle this by transparently releasing data and code (53) or by deliberately curtailing the power of the mechanism designer (e.g., by denying it memory) so that its policy is more amenable to simple description and display (79). However, building deep learning systems that are powerful and expressive and yet transparent and interpretable is an unsolved problem across AI research, and may represent a fundamental trade-off for practitioners and users (98, 99).

These ethical issues are not raised exclusively by the advent powerful AI but arise whenever data or algorithms are used for policymaking (100). Indeed, the rapid growth of algorithmic decision-making in spheres such as medicine, education, and justice has already raised important ethical concerns (72). Some worry that extrapolating this trend to public policymaking will usher in a dystopian era of “algocracy” in which societal rules are decided by machines rather than the votes of human citizens (101). For now, research into deep mechanism design remains largely at a proof-of-concept stage, and practitioners have been quick to point out that social or economic mechanisms designed by AI systems are not ready for deployment in the real world, especially where they touch on sensitive issues such as wealth redistribution or public health policy. Moreover, one could argue that empirical research into the consequences of AI-mediated mechanism design is important for understanding potential risks and harms and learning how to mitigate them. However, it is vital that research is conducted in a safe, responsible, and transparent way.

## Conclusions

Designing effective and popularly acceptable mechanisms is essential for the building strong economies, polities and societies. In this review, we have discussed new work in AI research that has begun to contribute to this endeavor. This work opens new opportunities for AI systems to act as “intelligent institutions” that propose new ways of optimizing human interaction for collective social benefit. However, we have also highlighted the importance of ensuring that deep learning is used for mechanism design in ethical and responsible ways so that any resulting social policies are considered legitimate and safe for human use.

## Data Availability

All study data are included in the main text.

## Footnotes

^*^This is equivalent to an ascending-price (or English) auction in which the price gradually rises until only a single buyer is willing to pay.

^†^An earlier version of the paper included data from human volunteers playing the game under the AI-derived tax regime, but results were somewhat equivocal (102).
